# Differences in the nutritional quality of improved finger millet genotypes in Ethiopia

**DOI:** 10.1038/s41598-023-48749-3

**Published:** 2024-01-03

**Authors:** Demeke Teklu, Dawd Gashu, Edward J. M. Joy, Elizabeth H. Bailey, Lolita Wilson, Tilahun Amede, Martin R. Broadley

**Affiliations:** 1 Center for Food Science and Nutrition, Addis Ababa University, Addis Ababa, Ethiopia; 2 Faculty of Epidemiology and Population Health, London School of Hygiene and Tropical Medicine, London, UK; 3 Rothamsted Research, West Common, Harpenden, Hertfordshire UK; 4 School of Biosciences, University of Nottingham, Sutton Bonington Campus, Loughborough, Leicestershire UK; 5 Alliance for a Green Revolution in Africa (AGRA), Sustainably Growing Africa’s Food Systems, Nairobi, Kenya

**Keywords:** Biotechnology, Plant sciences

## Abstract

Improved crop genotypes are constantly introduced**.** However, information on their nutritional quality is generally limited. The present study reports the proximate composition and the concentration and relative bioavailability of minerals of improved finger millets of different genotypes. Grains of finger millet genotypes (n = 15) grown in research station during 2019 and 2020 in Ethiopia, and replicated three times in a randomized complete block design, were analysed for proximate composition, mineral concentration (iron, zinc, calcium, selenium), and antinutritional factors (phytate, tannin and oxalate). Moreover, the antinutritional factors to mineral molar ratio method was used to estimate mineral bioavailability. The result shows a significant genotypic variation in protein, fat and fibre level, ranging from 10% to 14.6%, 1.0 to 3.8%, and 1.4 to 4.6%, respectively. Similarly, different finger millets genotypes had significantly different mineral concentrations ranging from 3762 ± 332 to 5893 ± 353 mg kg^−1^ for Ca, 19.9 ± 1.6 to 26.2 ± 2.7 mg kg^−1^ for Zn, 36.3 ± 4.6 to 52.9 ± 9.1 mg kg^−1^ for Fe and 36.6 ± 11 to 60.9 ± 22 µg kg^−1^ for Se. Phytate (308–360 µg g^−1^), tannin (0.15–0.51 mg g^−1^) and oxalate (1.26–4.41 mg g^−1^) concentrations were also influenced by genotype. Antinutritional factors to minerals molar ratio were also significantly different by genotypes but were below the threshold for low mineral bioavailability. Genotype significantly influenced mineral and antinutritional concentrations of finger millet grains. In addition, all finger millet genotypes possess good mineral bioavailability. Especially, the high Ca concentration in finger millet, compared to in other cereals, could play a vital role to combating Ca deficiency. The result suggests the different finger millet genotypes possess good nutrient content and may contribute to the nutrition security of the local people.

## Introduction

Finger millet (*Eleusine coracana* L.) represents one of the critical plant genetic resources for food security of populations from arid, infertile and marginal lands^1^. In the semiarid tropics of Eastern Africa, finger millet is the major staple food for millions of resource poor people^2^. Finger millet is adaptable to adverse agro-ecological conditions with minimal agricultural inputs (fertilizer, pesticides, and herbicides). It is also disease tolerant and is productive on marginal land where other crops can't be grown^3,4^.

Global finger millet production is not known because the crop is often grouped and reported with other millets. However, available reports show that motal production of finger millet is about 34 million tonnes worldwide^5^ and is estimated to represents about 12.8% (4.3 million tonnes) of all millet crops production^6^. In Ethiopia, finger millet is the sixth most important crops after teff (*Eragrostis tef* Zucc.), wheat (*Triticum aestivum* L.), maize (*Zea mays* L.), sorghum (*Sorghum bicolor* L*.*) and barley (*Hordeum vulgare* L.). An estimated 1.2 million tonnes of finger millet is produced in Ethiopia on 48 thousand hectares of land^7^. Nationwide, 1.5 million households are directly engaged in finger millet production and production has increased by 300% in the previous 20 years^7^.

During the last few decades, about 21 relatively high yielding genotypes of finger millet have been introduced in the cropping system, under the Ethiopian crop variety improvement programme. The national breeding program of finger millet has focused mainly on agronomic traits such as yields, drought tolerance and disease resistance^8^; however, there is limited information on nutritional quality. The present study evaluated the nutritional quality of finger millet grains of different genotypes in Ethiopia. Information on the nutritional quality of finger millet will help to design an advocacy work to increase its consumption and agricultural interventions that increase nutrient content on the edible portion of the crop.

## Results

Finger millet genotypes showed significant (p < 0.01) variation in mineral concentrations (Table 1). Greater variability was observed in Ca concentration ranging from 3540 (Axum) to 6117 (BKFM0010) mg kg^−1^. Bereda and BKFM0010 genotypes had the highest Ca concentration while Axum, Wama, Boneya, Bako-01 and Gudetu genotypes had the lowest Ca concentration (Table 1).

**Table 1:** Mineral concentrations (mg kg^−1^) of finger millet from Ethiopia as affected by genotypes.

Genotype	Fe	Zn	Ca	Se (µg kg^−1^)
Addis-01	47.7 ± 10.1^ab^	22.2 ± 2.5^abcd^	5140 ± 429^gh^	52.1 ± 19^ab^
Axum	36.3 ± 4.6^a^	19.9 ± 1.6^a^	3762 ± 332^a^	36.9 ± 9^a^
Bako-09	49.9 ± 13.4^b^	20.6 ± 4.1^ab^	3994 ± 231^abc^	45.1 ± 9^ab^
Bereda	44.3 ± 5.5^ab^	24.4 ± 1.9^cde^	5504 ± 412^hi^	51.5 ± 18^ab^
BKFM0010	48.2 ± 4.5^ab^	25.5 ± 3.4^de^	5893 ± 353^i^	60.9 ± 22^b^
Boneya	44.7 ± 12.3^ab^	21.4 ± 1.8^abc^	3977 ± 397^abc^	36.6 ± 11^a^
Diga-01	52.9 ± 9.1^b^	21.9 ± 1.8^abcd^	5193 ± 371^gh^	50.4 ± 15^ab^
Gudetu	48.6 ± 10.6^ab^	21.7 ± 2.6^abcd^	4237 ± 550^abcd^	52.4 ± 18^ab^
Gute	41.4 ± 7.5^ab^	20.3 ± 1.6^ab^	4315 ± 473^bcde^	43.5 ± 14^ab^
Meba	51.3 ± 10.9^b^	23.4 ± 2.5^bcde^	5008 ± 419^fgh^	48.7 ± 15^ab^
Paddet	48.7 ± 6.6^b^	26.2 ± 2.7^e^	4460 ± 381^cde^	43.6 ± 14^ab^
Tadesse	47.4 ± 5.8^ab^	25.3 ± 2.9^de^	4572 ± 311^def^	42.8 ± 12^ab^
Tesema	49.1 ± 7.7^b^	24.7 ± 3.4^cde^	4312 ± 259^bcde^	41.1 ± 10^ab^
Urji	49.7 ± 7.2^b^	23.9 ± 2.6^bcde^	4783 ± 395^efg^	47.7 ± 16^ab^
Wama	43.0 ± 9.6^ab^	20.4 ± 4.2^ab^	3782 ± 382^ab^	37.1 ± 13^a^

Significance at the p < 0.01 are represented with different letters.

Finger millet Zn concentration was significantly different (p < 0.001) between genotypes. Paddet and Axum genotypes showed the highest and the lowest grain Zn concentration, respectively (Table 1). Similarly, there was strong genotype influence on grain Fe concentration while moderate evidence (p < 0.01) was observed on grain Se concentration (Supplementary Table 1). Irrespective of locations the analysis of variance indicated that there was significant difference (p < 0.001) in antinutritional concentration among genotypes (Supplementary Table 2).

Phytic acid concentration of studied finger millet genotypes is presented in Table 2. Urji genotype showed significantly greater phytic acid and tannin concentrations. On the other hand, Addis-01, Boneya, Gute, Diga-01, Paddet and Gudetu genotypes had the lowest phytic acid concentrations (Table 2). In addition, Boneya, Diga-01, Gudetu, Gute and Meba genotypes had the lowest tannin concentrations (Table 2).

**Table 2:** Antinutritional concentration of finger millet from Ethiopia as affected by genotypes.

Genotype	Phytate (µg g^−1^)	Tannin (mg g^−1^)	Oxalate (mg g^−1^)
Addis-01	311.5 ± 2.9^a^	0.32^d^	3.39 ± 0.3^d^
Axum	319.6 ± 1.3^abcde^	0.25^bc^	3.15 ± 0.6^ cd^
Bako-09	330.6 ± 3.2^cdef^	0.31^d^	2.21 ± 0.3^ab^
Bereda	329.3 ± 2.9^bcdef^	0.28^c^	3.31 ± 0.2^d^
BKFM0010	348.5 ± 3.2^gh^	0.23^b^	1.81 ± 0.4^ab^
Boneya	313.5 ± 5.2^ab^	0.17^a^	3.39 ± 0.8^d^
Diga-01	317.1 ± 3.2^abcd^	0.17^a^	1.89 ± 0.3^ab^
Gudetu	323.5 ± 3.6^abcde^	0.17^a^	1.34 ± 0.2^a^
Gute	314.3 ± 4.8^abc^	0.16^a^	2.52 ± 0.3^bcd^
Meba	330.6 ± 4.3^def^	0.17^a^	2.21 ± 0.3^ab^
Paddet	323 ± 6.8^abcde^	0.24^b^	2.52 ± 0.3^bcd^
Tadesse	342.8 ± 2.6^fgh^	0.23^b^	2.37 ± 0.2^bc^
Tesema	341.4 ± 19.9^fgh^	0.24^b^	2.52 ± 0.3^bcd^
Urji	349.2 ± 3.5^h^	0.50^e^	1.89 ± 0.3^ab^
Wama	334.8 ± 4.5^efgh^	0.24^b^	2.21 ± 0.4^ab^

Significance at the p < 0.001 are represented with different letters.

The upper limit for intake of the antinutritional factors is 0.6 mg/kg of body weight for tannin^9^ and 50 mg/day of oxalate^10^.

Molar ratios of anti-nutritional factors to mineral concentration as proximate indicator for bioavailability are presented in Table 3. The molar ratio of phytate to Fe, phytate to Zn, phytate to Ca, phytate × Ca to Zn and oxalate to Ca was in the range of 0.51–0.71, 1.22–1.63, 0.004–0.005, 0.14–0.2 and 0.14–0.39, respectively (Table 3).

**Table 3:** Molar ratio of phytate to iron, zinc, calcium and oxalate to calcium in finger millet genotypes.

Genotype	Phytate:Fe	Phytate:Zn	Phytate:Ca	Phytate x Ca:Zn	Oxalate:Ca
Addis-01	0.55	1.39	0.004	0.18	0.30
Axum	0.74	1.59	0.005	0.15	0.38
Bako-09	0.56	1.59	0.005	0.16	0.25
Bereda	0.63	1.34	0.004	0.18	0.27
BKFM0010	0.61	1.35	0.004	0.20	0.14
Boneya	0.59	1.45	0.005	0.14	0.39
Diga-01	0.51	1.43	0.004	0.19	0.17
Gudetu	0.56	1.48	0.005	0.16	0.14
Gute	0.64	1.53	0.004	0.17	0.27
Meba	0.55	1.40	0.004	0.18	0.20
Paddet	0.56	1.22	0.004	0.14	0.26
Tadesse	0.61	1.34	0.005	0.15	0.24
Tesema	0.59	1.37	0.005	0.15	0.27
Urji	0.59	1.45	0.004	0.17	0.18
Wama	0.66	1.63	0.005	0.15	0.27

Cut off values: Phytate:Zn > 15, phytate:Fe > 1, phytate:Ca > 0.24, phytate × Ca:Zn > 200, and oxalate:Ca > 1.

Protein content of finger millet shows variation between genotypes (Table 4). Paddet shows significantly higher protein whereas BKFM0010, Gute, Bako-09 and Diga-01 varieties showed lower protein content. Variation in crude fibre content of finger millet varieties ranges from 1.44 to 4.63% (Table 4). BKFM0010 and Bako-09 varieties possess significantly higher and lower crude fibre content among the varieties, respectively. Total lipid and mineral concentrations were in the range of 1.05 to 3.81% and from 1.01 to 3.97% between the genotypes of finger millet, respectively (Table 4). The highest crude fat content was found in Gute while Paddet, Urji and Diga-01 showed significantly highest total mineral content. Variation in carbohydrate was in the range of 76.7 to 84.0%; Bako-09 and Axum were the genotypes with the highest carbohydrate content (Table 4).

**Table 4:** Proximate composition (g 100 g^−1^) of different finger millet genotypes.

Variety name	Protein	Crude fibre	Crude fat	Total ash	Carbohydrate
Addis-01	11.85 ± 0.53^ cd^	2.06 ± 0.04^b^	1.1 ± 0.02^a^	2.7 ± 0.25^d^	82.3 ± 0.51^hi^
Axum	11.68 ± 0.11^bc^	3.19 ± 0.12^d^	1.09 ± 0.02^a^	1.1 ± 0.08^a^	82.9 ± 0.14^ij^
Bako-09	11.09 ± 0.42^abc^	1.5 ± 0.05^a^	1.07 ± 0.02^a^	2.69 ± 0.1^d^	83.7 ± 0.35^j^
Bereda	12.99 ± 0.13^e^	2.3 ± 0.06^c^	1.9 ± 0.5^bc^	2.13 ± 0.01^c^	80.7 ± 0.35^ef^
BKFM0010	10.56 ± 0.41^a^	4.58 ± 0.06^ g^	1.67 ± 0.02^b^	3.32 ± 0.08^e^	79.9 ± 0.46^de^
Boneya	12.95 ± 0.39^e^	3.06 ± 0.08^d^	2.15 ± 0.06^c^	3.23 ± 0.07^e^	78.6 ± 0.54^b^
Diga-01	11.32 ± 0.39^abc^	2.19 ± 0.03^bc^	1.63 ± 0.07^b^	3.78 ± 0.03^f^	81.1 ± 0.33^ fg^
Gudetu	13.02 ± 0.11^e^	3.17 ± 0.09^d^	2.68 ± 0.1^d^	2.17 ± 0.04^c^	79 ± 0.32^bc^
Gute	11.07 ± 0.26^abc^	2.13 ± 0.11^bc^	3.77 ± 0.05^f^	1.11 ± 0.03^a^	81.9 ± 0.31^gh^
Meba	11.5 ± 0.37^bc^	3.8 ± 0.07^f^	2.17 ± 0.1^c^	1.61 ± 0.14^b^	80.9 ± 0.11^f^
Paddet	14.15 ± 0.58^f^	3.49 ± 0.09^e^	1.68 ± 0.12^b^	3.74 ± 0.19^f^	76.9 ± 0.29^a^
Tadesse	12.97 ± 0.23^e^	3.24 ± 0.1^d^	3.21 ± 0.08^e^	3.24 ± 0.1^e^	77.4 ± 0.25^a^
Tesema	12.71 ± 0.5^de^	3.18 ± 0.21^d^	2.64 ± 0.07^d^	1.6 ± 0.02^b^	79.9 ± 0.15^de^
Urji	11.46 ± 0.32^bc^	3.12 ± 0.03^d^	2.18 ± 0.06^c^	3.75 ± 0.09^f^	79.5 ± 0.43^ cd^
Wama	10.84 ± 0.31^ab^	3.55 ± 0.08^e^	1.62 ± 0.07^b^	1.6 ± 0.09^b^	82.4 ± 0.3^hi^

Significance at the p < 0.05 are represented with different letters.

## Discussion

Finger millet plays a major role in food and nutrition security for millions of resource poor smallholding farming communities^2^. Breeding programs often focus on agronomic traits such as yield, drought tolerance and disease resistance^8^. In addition, it is crucial to understand nutritional quality prior to genotype verification and seed release. The present study evaluated the nutritional quality (nutrient content, mineral relative bioavailability and antinutritional factors) of different genotypes of finger millet grains.

The present study revealed that finger millet genotypes are rich source of Ca. Sharma et al.^11^ and Kumar et al.^12^, also reported wide variation in finger millet Ca concentration in the range of 530 and 4540 mg kg^−1^ (n = 202 genotypes) and 720 to 4520 mg kg^-1^ (n = 113 genotypes), respectively. Similarly, finger millet Ca concentration ranges from: 1620 to 4870 mg kg^−1^ (n = 36 genotypes)^13^, 1505 to 4528 mg kg^−1^ (n = 26 genotypes)^14^, 2766 to 3319 mg kg^−1^ (n = 5 genotypes)^15^, 3341 to 3540 mg kg^−1^ (n = 3 genotypes)^16^, and 3180 to 6590 mg kg^−1^ (n = 12 cultivars)^17^. Varied concentration of Ca in finger millet was also reported by Patil et al.^18^, ranging between 9000 and 14,000 mg kg^−1^ (n = 37 genotypes). Previous studies partly associate variation in mineral accumulation in grains to specific genes in the plant. For example, Mirza et al.^19^ and Sharma et al.^11^ reported that *EcCBP* and *EcCIPK7* genes and the activities of CaX exchanger and calmodulin (CAM) proteins in finger millet resulted high Ca accumulation. They also reported that these two genes were highly expressed in high Ca genotypes compared to medium and low Ca genotypes^12,20^.

The present study revealed that finger millet Ca concentration was about 400% greater than that of other millets (Pearl, Proso, Foxtail, and Kodo) and 90% to 230% greater than other cereals such as wheat, barley, sorghum, maize, rice, and rye^21^. Genotypic variation in Zn concentration is in agreement with other similar studies. Puranik et al.^22^ reported finger millet Zn concentration between 10.2 and 26.6 mg kg^−1^ (n = 48 genotypes). Singh and Srivastava^23^ and Panwar et al.^15^ also experimented on finger millet and reported variation in Zn concentration ranging from 9.2 to 25.5 mg kg^−1^ (n = 16 genotypes) and 20 to 29.6 mg kg^−1^ (n = 5 genotypes), respectively.

Many reports on the genotypic variation in Fe concentrations in finger millet indicate concentrations in the range of 18.0 to 166.0 mg kg^−1^ (n = 106 genotypes)^16–18,22,24^. Similarly, Fe concentrations in the current study grain samples fall within this range. In addition, variation in Se concentrations as a result of genotypic differences are in agreement with those observed by Udeh et al.^25^ who found significant variation in Se concentration in the range 20 to 50 µg kg^−1^.

High accumulation of Fe and Zn in finger millet grains has been attributed to the regulation of potential key regulatory genes involved in Fe and Zn homeostasis particularly *EcFER1, EcIRT2, EcYSL2, EcZIP1* and *EcZTP29* genes^26^. Similarly, high concentrations of Se in finger millet has been also attributed to regulatory genes involved in Se homeostasis such as HOX4 and SPL genes^27^.

The analysis of variance indicates significant variation in phytic acid concentrations that are compared to those reported by Nakarani et al.^28^ which were in the range of 2108 to 3028 µg g^−1^ (n = 10 genotypes). Other studies also reported very wide variation (3363 to 14,020 µg g^−1^) in phytic acid concentration of finger millet^25,29^. The variation in current and previous studies is possibly influenced by genotype, soil, climatic factors and cropping season^30^. Phytate is the major storage (up to 82%) form of P in plants^31^ and every factor that affects plant P uptake also affects grain phytic acid concentration. For example, P become unavailable to plants in both acidic and alkaline soils but pH values between 6 and 7 are reported to be optimum for P absorption. On the other hand, P is subjected to iron and aluminium fixations at lower pH and by Ca at higher pH^32^. Both plant growth and P uptake are slow in the winter and release of the mineral from soil organic matter is apparently slow while in summer further decomposition of organic residues brings an increase in anion-exchangeable P and in soil P become soluble^33^.

Tannin concentrations were also lower than previously reported values. Nakarani et al.^28^ and Shibairo et al.^24^ reported values ranging between 3.4 and 5 mg g^−1^ (n = 10 genotypes) and 2.7 and 5.4 mg g^−1^ (n = 6 genotypes), respectively. Another study also reported a value of 1.6 ± 0.01 mg g^−1^ tannin concentration for finger millet^29^. The reason for lower tannin concentration in this study, besides genetic factors, could be attributed to the crop growing temperature which has been reported to influence the tannin concentration in cereals^34^, higher temperature might result in higher tannin concentration^24^.

Similar studies on oxalate concentrations in finger millet reports values ranging from 0.2 to 0.26 mg g^-1^ (n = 10 genotypes)^29^. The present study shows higher oxalate concentration which could arise for many reasons including: synergetic and antagonistic effect on oxalate from N and P, respectively^35^. Season/temperature also reported to influence oxalate accumulation^36^. For example, when nitrate is reduced, hydroxyl ions (OH^−^) are produced and the increased levels of OH^−^ may serve as a signal triggering the organic acid biosynthesis like oxalic acid to neutralize the excess levels of OH^−^^37^. There are more favourable growth factors prevailing in spring season which help higher metabolic rate of the younger tissues to synthesize oxalate^36^.

Phytates inhibit Fe, Zn and Ca absorption in to the human body system. Oxalate also inhibits Ca absorption by forming insoluble and indigestible complexes and additionally Ca competitively inhibits Zn absorption. The amount of these complexes and the molar ratio of phytate and oxalate to minerals may therefore affect bioavailability^38,39^. The molar ratio of phytate to the studied minerals shows that they are all less than cut-off values phytate:Zn is > 15 for low bioavailability, 5–15 for medium bioavailability and < 5 for high bioavailability^40^. The molar ratio of phytate x Ca:Zn of all finger millet genotypes in the present study was lower than the cut-off values of 200^41^ suggesting good Zn bioavailability. Similarly, the molar ratio of phytate:Fe, phytate:Ca and oxalate:Ca were lower than the cut-off values of > 1^42^, > 0.24^43^ and > 1^40^, respectively.

With respect to protein concentration, result of the present study is in agreement with previous experiment from Ethiopia reporting protein concentration in the range of 6.3 to 10.5% (n = 3 genotypes, 6 cultivars)^8^. Another study on finger millet indicates variation in crude protein content between 6.7 to 12.3% (n = 36 genotypes)^13^. Puranik et al.^22^, also analysed finger millet from East Africa and reported wide variation in crude protein ranging from 3.9 to 11.3% (n = 48 genotypes).

Carbohydrate content of finger millet in the present study shows variation between 76.7 and 84.1%. A previous study also reported that carbohydrate varies from 84.7 to 86.6% (n = 2 genotypes)^44^. Similar research on finger millet from Sri Lanka indicated variation in carbohydrate from 86.6 to 87.3% (n = 3 genotypes)^16^. Patil et al.^18^, Nakarani et al.^28^ and Shibairo et al.^24^, reported carbohydrate content in the range of 68.2 and 76.4% (n = 37 genotypes) and 71.9 and 76.4% (n = 10 genotypes), 75.6 and 78.5% (n = 6 genotypes), respectively.

Crude fibre content of finger millet genotypes ranges from 1.44 to 4.63% in the present study. which is in agreement with the study by Kaur et al.^17^, reporting that variation in crude fibre content of finger millet ranged between 3.2 and 5.8% (n = 12 cultivars). Patil et al.^18^ and Nakarani et al.^28^, also reported variation of finger millet’s crude fibre ranging from 3.7 to 4.2% (n = 37 genotypes) and from 3.1 to 3.8% (n = 10 genotypes), respectively. Similarly, previous studies reported variation in crude fibre content of different finger millet genotypes ranging from 3.1 to 5.6%^29,44^.

The present study shows that the total lipid content ranged from 1.1 to 3.8% between finger millet genotypes. Similar previous studies also reported wide variation in total lipid of finger millet from all over the world that ranges from 0.3 to 4.1% (n = 45 genotypes)^17,18,24,28,29,44^.

Total mineral content significantly varies between finger millet genotypes and ranges from 1.0 to 4.0%. Similar study on finger millet from Ethiopia shows 1.7 to 3.4% (n = 3 genotypes, 6 cultivars) variation in total mineral content^8^. Different researches around the globe shows that genotypically finger millet varies in their total mineral content, between 1.5% and 3.6%^16,17,23,44^.

## Conclusion

Our study shows that finger millet proximate composition, mineral and antinutrient content as well as mineral bioavailability significantly vary by genotype. The present study finger millet genotypes in general are good sources of Ca and protein, and a fair source of Fe and Zn. Moreover, all finger millet genotypes in present study exhibited excellent Zn, Fe and Ca bioavailability. Specifically, Bereda and BKFM0010 genotypes can be suggested for their highest mineral concentration and Paddet genotype for its highest protein for future breeding programmes. The highest concentration and relative bioavailability of Ca in finger millet could play a role in combating preeclamsia which is the second most cause of maternal mortality, Ca deficiency is the major factor of its occurrence^45^. Genotype, perhaps, significantly influences the minerals and anti-nutritional concentrations of finger millet. Even though finger millet has a high nutrient quality, use of finger millet in the daily diet is low^46^, suggesting the need for community nutrition education on the promotion of the nutritional benefit of finger millet and product development. Further investigations focusing on in vivo bioavailability testing of finger millet minerals are also strongly recommended.

## Study strength and limitation

The current study uses 15 out of 21 improved finger millet genotypes for experiment, all the field and laboratory experiments were replicated three times, about 270 finger millet samples were analysed in the laboratory, field experiments were repeated for two seasons and two locations, about 11 parameters were analysed in the laboratory. On the other hand, the current study uses the molar ratio of antinutrients to mineral to estimate bioavailability of minerals. This method is only a proxy indicator for minerals bioavailability.

## Material and method

### Field experiment

Out of a total of 21 improved new finger millet genotypes, 15 genotypes were obtained from seed maintainers (Dagi-01, BKFM0010 black grain colour, Urji white grain colour, Addis-01, Axum, Bako-09, Bereda, Boneya, Gudetu, Gute, Meba, Paddet, Tadesse, Tessema, Wama brown grain colour). Genotypes were improved for agronomic traits like high yield, disease resistance and stability^8^. Genotypes that are suitable for midland were selected for this study. The finger millet genotypes were grown in a randomized completed block design (RCBD) in field experiments at research stations, in two locations: Bako Agricultural Research Centre (9° 91′ 831″N 37° 42′ 492″E) and Gute sub site (9° 00′ 536″N 36° 38′ 243″E) to study the influence of genotype variability on the concentration and bioavailability of minerals over two seasons (during 2019 and 2020). Both sites are characterized as sub-humid midlands located between 1600 and 2300 m above sea level (masl) and receive an average annual rainfall of 800–1200 mm^45^.

### Agronomic management

The plot size was 3 m × 3 m, with gangway between plots being 1 m while distance between block and the border were 0.5 m each. The experiment was repeated in two growing seasons; 2019 and 2020. Seed was sowed in July and harvested in November. Planting was carried out by hand drilling at a seed rate of 15 kg ha^−1^. Each experimental plot had 40 cm inter-row spacing. Fertilizers, NPS (131 kg ha^−1^) was applied at sowing and urea (54 kg ha^−1^) was applied after 45 days at first weeding. Each plot was weeded at least six times by hand and no pesticide or herbicides was applied.

### Sample collection and preparation

After obtaining permission from the Ethiopian Agricultural Research Institute, crop samples were collected from the farm and prepared in the laboratory following the method as described in Gashu et al.^46^. Briefly, matured and dried finger millet crop fingers were collected from each plot using scissors. The crop samples were hand threshed in the laboratory to produce approximately 1 kg of grain before whole-grain samples were packed in paper bags and allowed to air dry. The grain samples were then ground using a stainless-steel coffee grinder, which was wiped clean before use and after each sample with a non-abrasive cloth. All preparations were done away from sources of soil and dust contamination. A 20 g subsample (following a representative coning and quartering system) of the ground finger millet was shipped to the University of Nottingham, UK for mineral analysis. The use of plants in the present study complies with international guidelines.

### Mineral analysis

Ground finger millet grain samples were acid digested in a hot plate as described in Gashu et al.^46^. Briefly, about 0.2 g of sample was weighed into digestion tubes and placed into a heating block (Multicube 48, Anton Paar Ltd, UK). Concentrated HNO_3_ (8 mL, trace metal grade, Fisher Chemical, USA) was added to each tube and left for 30 min at room temperature. The samples were then heated for 2 h at 115 °C and left to cool before dilution to 50 mL using MilliQ water (18.2 MΩ cm; Fisher Scientific). A further 1 in 10 dilution was undertaken immediately prior to analysis by inductively coupled plasma-mass spectrometry (ICP-MS) (Thermo Fisher Scientific, Bremen, Germany). A certified reference material (CRM, Wheat 1567b, National Institute of Standards and Technology, Gaithersburg, MD, USA) was used to determine % recovery. Operational blanks (n = 20) were analysed at the same time to determine the limit of detection (LOD) for each element.

### Anti-nutritional factors analysis

Phytic acid was analysed using the Wade Reagent method, after Latta & Eskin^47^ and later modified by Vaintraub & Lapteva^48^. For extraction of phytic acid 0.2 g of  flour samples were centrifuged at 3000 rpm for 1 h after adding 10 mL of 0.2 M HCl. Then 3 mL of the supernatant and 2 mL of Wade solution were added and samples were shaken to mix. Absorbance was measured at 520 nm using a UV–VIS spectrophotometer (Lambda 950, PerkinElmer, Waltham, USA) and the amount of phytic acid was calculated.

Oxalate in the flour samples was determined following the method of the Association of Official Analytical Chemists^49^. Briefly, 1 g of sample was weighed into a 100 mL conical flask before 75 mL of 3 M H_2_SO_4_ was added and the solution was mixed for about 1 h before filtering. The filtrate was collected and titrated against hot (80–90 °C) 0.1 M KMnO_4_ solution to the point when a faint pink color appeared that persisted for at least 30 s. The concentration of oxalate in each sample was obtained using the assumption that 1 mL 0.1 M KMnO_4_ = 0.006303 g oxalate^49^.

Tannin was determined using the vanillin-HCl assay method^50,51^. Briefly, 10 mL of 1% HCl in methanol was added to 1 g grain flour in a screw capped test tube and placed on a mechanical shaker for 24 h at room temperature. The tube was centrifuged at 1000 g for 5 min and 1 mL of supernatant was removed and mixed with 5 mL of vanillin-HCl reagent. Absorbance at 500 nm was measured after 20 min.

### Phytate and oxalate to mineral molar ratio calculation

The inhibitory effect of dietary phytate on the bioavailability of Fe, Zn and Ca, and oxalate on Ca bioavailability was determined through calculation of molar ratios (phytate:Fe, phytate:Zn, phytate x Ca:Zn and phytate:Ca and oxalate:Ca) and the millimoles used were 660 mg/mmol for phytate, 55.845 mg/mmol for Fe, 65.4 mg/mmol for Zn, 40 mg/ mmol Ca and 88.019 mg/mmol for oxalate^38,39^. Phytate:Zn > 15^40^, phytate:Fe > 1^42^, phytate:Ca > 0.24^43^, phytate × Ca:Zn > 200^40^, and oxalate:Ca > 1^40^ were used as cut-offs. Samples with molar ratio values higher than the cut-off values were considered less bioavailable.

### Proximate composition analysis

#### Crude protein

Crude protein content of samples was quantified by Kjeldahl methods^49^. Briefly, 0.5 g of powder sample was weighed into tecator tube and digested by heating at 370 °C for 3 h in the presence of 6 mL mixed sulfuric acid (H_2_SO_4_), 3.5 mL hydrogen peroxide (H_2_O_2_), 3 g of catalyst mixture: potassium sulphate (K_2_SO_4_) and copper sulphate (CuSO_4_). After digestion was completed, the clear solution was cooled for 30 min. After cooling, it was distilled by steam distillation with 25 mL of 40% of sodium hydroxide (NaOH) and the ammonium is released as a form of ammonia (NH_3_). Finally, the condensed NH_3_ is trapped by 1% boric acid and titrated by 0.1N hydrochloric acid (HCl). The nitrogen content was estimated by titration of the borate anion formed with 0.1N HCl. The amount of Nitrogen was calculated using the following equation:

1 $${\text{Nitrogen }}\% \, = \, \left( {{\text{V }} \times {\text{ N }} \times { 14 } \times { 1}00} \right) \, / \, \left( {{1}000 \, \times {\text{ Wo}}} \right),$$

where; V- Volume of HCl consumed to the end point of titration, N- The normality of the HCl used, Wo- Sample weight on dry matter basis, 14- The molecular weight of the atomic nitrogen

2 $${\text{Protein }}\% \, = {\text{ Nitrogen }}\% \, \times { 5}.{54}{\text{.}}$$

#### Crude fat

Crude fat was determined by Soxlet extraction method^49^. Extraction cylinders were measured (W1) after cleaned and dried in an oven at a 105 °C for 1 h. The bottom of the extraction thimbles were covered with a layer of fat free cotton and approximately 2 g of powder samples were measured in thimbles and covered with cotton layer (W). The thimbles were put in the extraction chamber. Extraction cylinders were filled with 50 ml of ether and moved into the heating plank. The extraction was run for about 4 h and then the extraction cylinders were disconnected and put in a drying oven at 70 °C for about 30 min. The cylinders were taken out of the oven and cooled in a desiccator for 30 min and the weight of cylinders were measured (W2). Finally the fat content of the samples were determined using the following equation:

3 $${\text{Crude}}\,{\text{fat }}\% \, = \, \left[ {\left( {{\text{W2}} - {\text{W1}}} \right)/{\text{W}}} \right] \, \times { 1}00.$$

#### Crude fibre

Two crucibles were cleaned and dried with 1 g celite in an oven at 105 °C for 1 h. Approximately 1 g of flour sample was weighed into pre-dried crucible (W1). Then, 200 mL of 1.25% H_2_SO_4_ was added to each crucible and left to boil for 37 min. The acid was drained using a vacuum pump after 37 min and the samples were cooled for 5 min, washed with distilled water. Then, 200 mL of 1.25% NaOH solution was added into each crucible and let to boil for 37 min. The base was drained using a vacuum pump and washed with distilled water. Crucibles containing residue were dried at 130 °C for 2 h and cooled in a desiccator and weighed (W2). The residues were ashed in a muffle furnace at 550 °C for 3 h and left to cool down to below 250 °C before removing from the furnace. The crucibles were cooled in a desiccator to room temperature and their weight were measured using analytical balance (W3)^49^.

4 $${\text{Crude fibre }}\% \, = \, \left[ {\left( {{\text{W2}} - {\text{W3}}} \right)/{\text{W1}}} \right] \, \times {1}00.$$

#### Total ash

Total ash content was determined by following a method as described by the Association of Official Analytical Chemists^49^. Briefly, porcelain crucibles were cleaned, dried and their weight were measured (M1) after being cooled in a desiccator for 30 min. Approximately 2.5 g of flour sample were measured in each crucible (M2) and charred on a hot plate under a fume hood until the smoke ceased down. Then the samples were ashed in muffle furnace at 550 °C for 5 h and left to cool down to below 250 °C before removing from the furnace. The crucibles were cooled in a desiccator to a room temperature and their weight were measured (M3) and the ash content was determined by using the following equation:

5 $${\text{Total}}\,{\text{ash }} = \, \left[ {\left( {{\text{M3}} - {\text{M1}}} \right) \, / \, \left( {{\text{M2}} - {\text{M1}}} \right)} \right] \, \times { 1}00.$$

### Statistical analysis

Data were analysed using SPSS software version 20. The data are presented as mean ± standard deviation (SD). Analysis of variance (ANOVA) was calculated to compare nutrient levels and concentrations of antinutritional factors across finger millet genotypes. Genotype was treated as fixed effect whereas block within farm and location were treated as random effects. The variance component for random effects was checked to get an idea of how important they might be relative to each other. p < 0.05 was considered significant.

### Supplementary Information

Supplementary Tables.

## Data Availability

The data generated in this study is available upon request from the corresponding author.
