# Supplementary material for: Differences in the nutritional quality of improved finger millet genotypes in Ethiopia
Source: Sci Rep. 2024 Jan 3;14:460. doi: 10.1038/s41598-023-48749-3 (PMC10764915; doi:10.1038/s41598-023-48749-3)
Supplement: Supplementary file 1 — Supplementary Tables. [file 41598_2023_48749_MOESM1_ESM.docx]

Supplementary Table 1: Analysis of variance of the effect of finger millet genotype on mineral concentration

|  | Sum of Squares | Mean Square | Df | F | Sig. |
| --- | --- | --- | --- | --- | --- |
| Fe | 3053.161 | 218.083 | 14 | 2.825 | .001** |
| Zn | 748.590 | 53.471 | 14 | 7.229 | .000** |
| Ca | 69304620.088 | 4950330.006 | 14 | 32.998 | .000** |
| Se | .008 | .001 | 14 | 2.604 | .002* |

Significance code: ** < 0.001; * < 0.01

Supplementary Table 2: Analysis of variance on effect of finger millet genotype on finger millet antinutritional concentration

|  | Sum of Squares | Mean Square | Df | F | Sig. |
| --- | --- | --- | --- | --- | --- |
| Phytate | 8938.665 | 638.476 | 14 | 15.610 | .000** |
| Tannin | .422 | .030 | 14 | 193.473 | .000** |
| Oxalate | 21.807 | 1.558 | 14 | 12.375 | .000** |

Significance code: ** < 0.001
